# The Mouth to Mouth vs. the Marshall Hall Method in Asphyxia Neonatorum*Read before the Cincinnati Medical Society.

**Published:** 1859-12

**Authors:** A. T. Keyt

**Affiliations:** Walnut Hills, Ohio


*The Mouth to Mouth* vs. *The Marshall Hall Method in Asphyxia Neonatorum.** By A. T. Keyt, M. D., Walnut Hills, Ohio.

Soon after the announcement by Marshall Hall of his "Ready Method" in asphyxia, communications appeared in the journals containing reports of cases to the effect that the process in them had been successfully employed. Like reports have since greatly multiplied, consisting of some cases of asphyxiated adults, but for the most part of asphyxiated new-born children, in which resuscitation followed more or less quickly the application of the new method. In these cases, the process would seem to have accomplished all that could be desired; and if, in accordance with the expressed view of some of the reporters, the children could not have been restored by any other means, then the question is settled, a true advancement has been made in the discovery of a treatment by which children that were doomed under the former methods may be saved. Nevertheless, for one, I am not thus sanguine, and propose the question: Which method,

---

* Read before the Cincinnati Medical Society.

the *Marshall Hall* or the *mouth to mouth*, is the more entitled to our confidence in the asphyxia of new-born infants?

It may be remembered that the case of the asphyxiated new-born child is not just parallel with that of the asphyxiated adult. The first has never respired. The chest has never been expanded; the air vesicles have never been opened. The chest and lungs then do not possess that elasticity or resiliency which would be so important an element in successfully carrying on the "rotation process." It would be difficult to understand how, under it, the first expansion of the lungs could take place; when the child is turned on its face, the lungs being already compressed, the capacity of the chest could be thereby but little, if any, diminished; and when turned upon the side and a little beyond, as directed, it could be but little, if any, increased. Whether here there be any ingress and egress of air has yet to be determined by experiment. This test, instituted, as is well kown, by Marshall Hall at St. George's Hospital, was in reference to adults. This peculiarity then, of the new-born child can but constitute a serious impediment to the successful performance of the *rotation process.* But it possesses characteristics which render it a very suitable subject for the mouth to mouth operation: its small size admits of its being placed in any position that may best suit the convenience of the practitioner; its small mouth is easily encompassed by his mouth, and the force of his breath is entirely sufficient to expand its delicate lungs; and the child, accustomed as it has been to imperfectly arterialised blood, is readily quickened into life by contact with its air vesicles of air no purer than the breath of the practitioner; moreover, no real difficulty is here experienced in the falling back of the tongue.

If the two processes be compared in reference to the qualities *ready*, *easy of performance*, and *effectual in the purpose*, it is plain that the old plan is as *ready* as the new, since by it the child is treated "*instantly and on the spot;*" that *ease of performance* pertains almost as much to the one as to the other (since no apparatus or complicated maneuvre is required in either case), and if there be here a slight difference in favor of rotation, it is not such as to influence the mind of the practitioner in deciding which process to pursue.

The great point is, which is the most effectual in the purpose

—the accomplishment of artificial respiration. Under the new method, there are no indications whereby it may be known whether the air enters the lungs or not, until waiting for evidences of increased vitality on the one hand, or the cessation of all signs of life on the other. Under the old method, there are *direct immediate* and *unmistakable signs that respiration is being effected:* the thorax expands, *the air is heard and felt to enter the lungs;* the thorax contracts, *and the air is heard and felt to escape.* Such evidences are conclusive; when present there is respiration, and there is no occasion that they should over be wanting.

I propose now to consider the question in the light of cases occurring in practice. I will take it for granted that the cases published as favorable to the new method are familiar to the profession, so as to render it unnecessary to reproduce any of them here.

Mrs. C——, third confinement, July 22, 1855. I found the child presenting by the feet. Labor steadily advanced till the body of the child was expelled, when an interval of at least a quarter of an hour elapsed before the head followed, during which time the circulation through the cord was completely obstructed. The child was born appearently dead, though the hand applied to the chest detected a very feeble pulsation of the heart. Artificial respiration by the *mouth* to *mouth* process was immediately resorted to, under which the action of the heart became more vigorous. After persevering for several minutes, there was a feeble respiratory gasp; then, after a considerable interval, there was another gasp. These respiratory acts continued to be repeated at long intervals, artificial respiration in the mean time being kept up and the circulation thereby maintained. An hour passed before the child could maintain its respiration unassisted. In the course of another hour there was a perfect restoration of vitality.

Mrs. B——, multipara. Confined November 6, 1855. One week previously she was taken with flooding, which continued more or less until true labor pains set in; after which there was no more flow. In this case there was prolapsus of the cord before the head, and my efforts were unavailing to keep it above the brim. The usual consequences followed: the cord was compressed, its pulsations grew faint and finally ceased; the child was born to all appearance lifeless. Examination, however, revealed the important fact that the heart was still pulsating, though so feebly as to be just perceptible, and so slowly that the lengthened interval excited my apprehension that each beat would be the last. With the loss of as little time as possible, I commenced artificial respiration (by the usual method), the effect of which was soon manifested on the circulation; the action of the heart was increased until it came up to the standard of a healthy breathing infant; the surface changed from pallor and lividity to the healthy complexion.

But it seemed that vitality was to be maintained only so long as I persevered in inflating the lungs; for on suspending the operation, there was no spontaneous respiratory effort, and the circulation became speedily feeble. However, after keeping up the process at least half an hour, I was greeted by a respiratory gasp. There was a recurrence of gasps at first at long intervals, and afterwards more frequently, until respiration was established.

These cases, I am aware, contain nothing peculiar. They are merely examples of asphyxiated new-born infants relieved efficiently by the method which the profession has been taught to recognize as the *sine qua non* in such conditions. I relate them that they may be placed in apposition with the cases published in the journals as favorable to the "Marshall Hall Method." And if it be claimed that in the latter cases resuscitation could not have been brought about by other than the "Ready Method," I think it may be equal propriety, at least, be claimed that my cases could not have been restored by other than the *mouth to mouth* method. Indeed, my firm conviction is, that when these births occurred, had I known of this new method, and resorted to it, and trusted it to the exclusion of the old process, these children would never have breathed. The asphyxia was so profound, and the tendency to complete death so rapid, that nothing less than the prompt and free exposure of the air vesicles to the air, as obtained by this process, would suffice.

I shall now reproduce some cases in which both methods were empolyed.

Mrs. C——, primipara; foot presentation, with descent of the cord, which latter was pulseless. I had no expectation of saving the child, and announced to the friends that it would be born dead. With no more delay than the average, delivery was completed. The child was small, and evidently not less than six weeks premature. There was nothing about it to indicate to the casual observer the presence of life, but by a careful examination the heart was ascertained not to have ceased its action—the pulsations were just perceptible to the ear. I entered at once upon the task of resuscitation. *At least one hour elapsed before the child gave a gasp*, and two hours before it could be left to do its own breathing. My dependence was upon the *mouth* to *mouth* process: by it I found no difficulty in controlling the circulation! It seemed as though the heart's action might have thus been maintained indefinitely. The "Marshall Hall Method" was tried, but the results were negative; under it pallor and lividity would return to the surface, and the circulation grow gradually more and more feeble, until the heart's action would plainly have soon ceased, had it not been timely aroused by a more *ready* and *efficient* method. Several times did I alternate the new method with the old, and jnst as often did I witness the same striking contrast of phenomena.

Mrs. L——, multipara; vertex presentation; child large and asphyxiated; restored by artificial respiration (the ordinary method). The "Marshall Hall Method" was found insufficient.

Mrs. T——, multipara; child delivered before my arrival by a breach presentation. It was gasping feebly and at distant intervals; pulsations of the heart very feeble and slow; color pale and livid. Restored by artificial respiration performed in the usual way. The "Ready Method" again unvailing. Though not stated in the record of these two cases, which is too brief, I distinctly remember that I gave the plan what would be considered a fair trial, and when under it I saw the child was evidently sinking, instead of evincing increased vitality, I felt it my duty no longer to trust it.

Cases such as these, certainly have a distinct and significant bearing upon this question. The processes stand side by side in the identical case; the one is found wanting—the other gives prompt and decided results; under this the circulation rises, the color changes, the child is reviving, and I am encouraged to persevere—under that the heart beats more and more freely, the lividity deepend, the child is sinking, and I am warned to desist.

I confess, then, to having great confidence in the old method of treating asphyxiated new-born children. I believe it is adequate to resuscitate wherever resusciation is possible. Give me a beating heart, however feeble and slow the action, and I expect to resuscitate the child; but if motion there has absolutely ceased, I am convinced that the best exertion in the use of any means would be in vain. The doctrine of Benjamin Brodie. that "if that action of the heart by which the circulation is maintained once cease as a consequence of the suspension of respiration, it can never be restored," has not been disproved. Dr. Marshall Hall proposed to put "this dogma" to the test of experiment, but unfortunately he died before carrying out the purpose,

It is certainly true that the new-born child bears the deprivation of air with peculiar tolerance. This fact I would use as an incentive to institute and preserve in efficient means at resuscitation. I would not use it as an apology for the employment of temporizing means of treatment. for there is a point beyond which it is not safe to leave the child. When the babe is born presenting no sign of life excepting as ascertained by a physical examination of the cardiac region, where the heart is found beating with a barely perceptible pulsation, then no time is to be lost· "The life-giving process" must be instantly commenced; a moment's delay in inflating the lungs may seal the infant's fate. An extremity like this calls for a remedy prompt and sure; such remedy is found in artificial respiration by the mouth to mouth method.

Rotation, I will grant, is entitled to a position as a means of resuscitation. It is easy to conceive that it would be a good exciter of respiration, and that after the lungs had once been expanded, sufficient air might be admitted by it to insure restoration. But in cases of profound asphyxia, this is not the remedy; its position is subordinate to that process in which the air is surely, quickly and freely admitted to the lungs.—*Cincinnati. Lancet & Observer.*

